# CD161 and LLT1 expression in immune cell populations of Hodgkin lymphoma tumor microenvironment

**DOI:** 10.3389/fmed.2026.1914176

**Published:** 2026-08-19

**Authors:** Anwar Rjoop, Rania Al-Samama’h, Laith Al-Eitan

**Affiliations:** 1 Department of Pathology and Microbiology, Faculty of Medicine, Jordan University of Science and Technology, Irbid, Jordan; 2 Department of Medical Laboratory Science, Faculty of Applied Medical Science, Jordan University of Science and Technology, Irbid, Jordan; 3 Department of Biotechnology and Genetic Engineering, Jordan University of Science and Technology, Irbid, Jordan

**Keywords:** background immune cells, CD161, Hodgkin lymphoma, immunohistochemistry, immunotherapy targets, LLT1, tumor microenvironment

## Abstract

**Introduction:**

Hodgkin lymphoma (HL) tumor microenvironment (TME) is composed of an abundant immune cell population, with relatively few neoplastic Reed-Sternberg (RS) cells. These immune cells fail to initiate an anti-tumor response or prevent immune evasion. Both Lectin-like transcript 1 (LLT1) and CD161 are an immune checkpoint axis that modulates immune cells’ responses in chronic infections, cancer, and autoimmune diseases. However, their expression within the HL TME is not well studied.

**Methods:**

This is a retrospective observational study that was conducted using archived formalin-fixed paraffin-embedded (FFPE) lymph node tissues from tertiary care center in Northern Jordan. A total of 56 HL cases and 60 reactive lymphoid tissue controls (benign lymph nodes and tonsils) were included. Immunohistochemistry (IHC) was performed to assess LLT1 and CD161 expression in background immune cells (BICs). The primary outcomes were the expression levels and localization patterns of LLT1 and CD161. Associations with clinicopathological variables were analyzed using Chi-square, Mann–Whitney U, Kruskal–Wallis, and Spearman correlation tests. Survival analysis was performed using the Kaplan–Meier method.

**Results:**

A significant correlation was observed between LLT1 and CD161 expression in the tumor microenvironment, both in the frequency of positive background immune cells) (BICs (*p* = 0.030) and in their expression localization patterns (*p* = <0.001). An enrichment of CD161-expressing immune cells was significantly associated with mixed cellularity cHL (*p* = 0.047) and with advanced-stage disease (Stage III–IV) (*p* = 0.010). In contrast, EBV-negative cases exhibited significantly higher LLT1-positive immune cell infiltration than EBV-positive cases (*p* = 0.036), whereas CD161 expression was not associated with EBV status (*p* = 0.746).

**Conclusion:**

LLT1 and CD161 expression in HL BICs may suggest an immune-to-immune interaction within TME. CD161 enrichment in advanced disease and the mixed cellularity subtype highlights their potential role in sustaining immune recruitment and retention. Thus, LLT1 and CD161 expression patterns position them as potential targets for immunotherapy in HL.

## 1 Introduction

Hodgkin lymphoma (HL) is a B-cell lymphoma characterized by the presence of Hodgkin/Reed-Sternberg (HRS) cells in tissue biopsies (1, 2). Its tumor microenvironment (TME) is considered one of the most inflammatory TMEs, and malignant cells represent an extremely low fraction of it (3). Despite the high immune cell infiltration, these immune cells play a role in the HL immunosuppressive niche to promote Reed-Sternberg (RS) cells’ survival, proliferation, and evasion, through chemokines, cytokines, growth factor secretion, and interactions with immune checkpoints (3–9).

CD161 and its ligand, Lectin-like transcript 1 (LLT1, encoded by *CLEC2D*), are emerging immune checkpoint molecules that play a critical role in modulating both innate and adaptive immune responses within the TME. While CD161 is mainly expressed on T cells and Natural Killer (NK) cells, LLT1 expression is induced on various cells, including tumor cells and antigen-presenting cells (APCs) (10–14). The CD161/LLT1 axis acts as an inhibitory checkpoint in cancer, facilitating tumor immune evasion (14–17). Both CD161 and LLT1 were found to be highly expressed in different types of cancers, including triple-negative breast, prostate, glioblastoma, colorectal, lung, and other cancers (11, 15, 16, 18–20). Blocking the CD161/LLT1 interaction using anti-LLT1 or anti-CD161 antibodies has been shown to enhance NK-mediated killing and restore T-cell-mediated cytotoxicity against cancer cells in preclinical models (15, 21). The CD161/LLT1 axis is, therefore, a potential new therapeutic target for cancer types that do not respond to traditional immune checkpoint inhibitors like PD-1/PD-L1 blockers. Currently, the first-in-human Phase 1 clinical trial of ZM008 (NCT06451497), a monoclonal antibody disrupting the LLT1-CD161 pathway, is underway to evaluate its safety and efficacy in advanced solid tumors (22). Another clinical trial evaluating IMT-009 (NCT05565417), a monoclonal antibody targeting the CD161 receptor, is recruiting patients with advanced solid tumors and specific B-cell non-Hodgkin lymphoma (21). While these ongoing clinical investigations have not yet formally expanded to include Hodgkin Lymphoma (HL), their expression in HL remains poorly identified (10, 21, 23, 24). Specifically, in Jordan, where HL incidence rates exceed those reported in Asian and Western countries (25).

While our previous work evaluated LLT1/CD161 expression in Reed–Sternberg cells (26). The immunobiological significance of this axis in the background immune compartment of HL remains undefined. We therefore characterized LLT1 and CD161 expression in HL-associated immune cell infiltrates and explored their possible associations with clinicopathological features, EBV status, and immune cell composition. By integrating the study of both malignant cells and the surrounding immune microenvironment, our work offers a broader perspective on the potential immunological and therapeutic relevance of CD161 and LLT1 in Hodgkin lymphoma.

## 2 Materials and methods

### 2.1 Human samples and data collection

Overall, 116 FFPE tissue samples were included in this study. Sixty FFPE tissue samples of patients diagnosed with Hodgkin lymphoma (before treatment) were retrospectively collected from the pathology department of King Abdullah University Hospital (KAUH) archives, a major hospital serving the northern region of Jordan, between December 1st, 2024, and January 12th, 2025. Further details about the cohort were shown in the Supplementary Table 1. Inclusion criteria for our cases: diagnosed with HL, females and males, any age (children and adults), tissue before treatment, and availability of adequate FFPE tissue blocks suitable for immunohistochemistry (IHC) staining. Exclusion criteria: non-HL diagnosis, post-treatment tissue, and FFPE blocks with insufficient tissue for sectioning or poor preservation. A total of four samples were excluded from the final analysis due to inadequate FFPE tissue, leaving 56 samples for full IHC evaluation.

Additionally, 60 FFPE samples of reactive lymphoid tissues, including benign reactive lymph nodes and tonsils, were used as a control to assess CD161 and LLT1 expression (26). For negative controls, the primary antibody was omitted.

Moreover, to confirm diagnoses we reviewed each FFPE tissue block corresponding to hematoxylin & eosin (H&E) staining and reviewed archived diagnosis markers, such as CD15, CD30, PAX5, CD20, MUM1, and CD3, for the cohort when available.

This study was a retrospective analysis of archived samples, clinical data and results did not influence patients’ management or treatment.

### 2.2 Immunohistochemistry (IHC) procedure

IHC staining for LLT1/CD161 was performed as previously described (26). Briefly, the FFPE blocks were manually sectioned by microtome with a thickness of 3–4 μm then mounted on positively charged slides (Superfrost® Plus). To enable and strengthen tissue adherence they were placed in a drying oven for 30 min at 87 °C. Finally, uploaded to the fully automated staining Ventana BenchMark ULTRA system (Ventana Medical Systems, Tucson, AZ, USA), in which the sectioned tissues were deparaffinized, subjected to antigen retrieval, and incubated with primary antibodies specific to CD161 (anti-KLRB1/CD161 mAb clone 3D8F5, Proteintech, Catalog No. 67537-1-Ig, RRID: AB_2882756, 1:1,000), EBV (anti-EBV/LMP-1 mAb clone S20-D, MyBioSource, Catalog No. MBS684063, 1:100), LLT1 (anti-CLEC2D mAb clone 4C7, Abnova, Catalog No. H00029121-M01, RRID: AB_463941, 1:50), and D8 (anti-CD8 mAb clone 4B11, MyBioSource, Catalog No. MBS215242, RRID: AB_10887243, 1:50). For detection of the primary antibodies, the OptiView DAB IHC Detection Kit (Ventana) were used. The slides were counterstained with Hematoxylin II and treated with bluing reagent to reinforce nuclear contrast.

For scoring CD161 and LLT1 expression in background immune cells (BICs), the percentage of positive cells was used. Then, they were sub-grouped into negative (0% positive cells/HPF), low (≤ 50% positive cells/HPF), and high (> 50% positive cells/HPF) frequency groups.

To characterize the immune cells composition within HL tumor microenvironment in the study cases, lineage stains were performed. CD8 IHC staining was performed for all cases (n = 56). Archived diagnostic slides for CD3 and CD20 were available for only 34 patients in our cohort, and these cases were used to assess the composition of immune cells within the HL tumor microenvironment.

T and B cell markers were scored as the percentage of positive cells based on a visual estimation of CD3-and CD20-positive lymphocyte populations per high-power field (HPF) across representative areas and reported into conventional semi-quantitative categories as: low ≤ 10% of positive cells/HPF, moderate >10–50% of positive cells/HPF, high > 50% of positive cells/HP.

CD8^+^ T-cell frequency was estimated as the percentage of CD8^+^ cells relative to the total CD3-positive T-cell population for each patient, based on visual assessment of representative high-power fields (HPFs). Cases were subsequently classified using the same semi-quantitative categories as those applied for CD3 and CD20: low (≤10%), moderate (>10–50%), and high (>50%) CD8-positive T cells.

For CD4 assessment, archived CD4-immunostained slides were available for only a limited number of cases (*n* = 4). For the other cases the CD4^+^ T-cell frequency was estimated as the proportion of CD3^+^CD8^−^ T cells. Cases lacking archived CD3 data were excluded from the CD4^+^ T-cell analysis; consequently, CD4^+^ T-cell frequency was assessed in 34 cases only.

EBV expressions were assessed by the percentage of positive cells based on a visual estimation of positive or negative membranous and cytoplasmic staining of RS cells. Cases were considered positive when ≥1 RS cell demonstrated distinct LMP1 immunoreactivity, consistent with latent EBV infection.

The scoring was performed independently by two observers, including a pathology consultant (AR) and a researcher (RS), both were blinded to the clinical data. The interobserver agreement was > 90%; any discrepancies were resolved through joint review using a multi-headed microscope.

### 2.3 Statistical analysis

The data were analyzed using IBM SPSS Statistics version 27.0 (IBM Corp., Armonk, NY, USA). Q–Q plots and Shapiro–Wilk tests were used to assess the distribution of the continuous variables, which showed that they were statistically significant (*p* < 0.05) not normally distributed. The chi-square test (or Fisher’s exact test to confirm the assumptions) was used to assess associations between categorical variables, such as marker frequency groups (high versus low). To evaluate associations between ordinal variables, Spearman’s rank correlation coefficient was applied to the CD161 and LLT1 frequency groups. The Mann–Whitney U and Kruskal-Wallis tests were applied to compare non-parametric variables between independent groups, such as CD161and LLT1 percentages of positive immune cells in EBV-positive vs. EBV-negative cases, HL stages, and subtypes.

Kaplan–Meier analysis to investigate the effects on overall survival (OS) and progression-free survival (PFS). The OS period for each patient was calculated from the date of diagnosis to the date of death, or the last follow-up date, or any last encounter with the patient in any hospital department available in the patient’s hospital e-records. The PFS period for each patient was calculated from the date of diagnosis to the relapse or death date or the last progression-free follow-up date. Follow-up data were available for 46 patients; 41 patients were alive, and five had died at the time of data collection in April 2025. Ten patients were excluded from the survival analysis due to missing follow-up or survival data.

This study is exploratory in nature, and the findings were interpreted considering the limited cohort size (*n* = 56). Cases with missing or insufficient samples were excluded from statistical comparisons. and *p*-values < 0.05 (2-sided) were considered statistically significant.

## 3 Results

### 3.1 Patient cohort: demographics and clinical characteristics

In this study, a total of 56 patients were diagnosed with Hodgkin lymphoma (HL), including 33 males (58.9%) and 23 females (41.1%). The median age at diagnosis was 30 years and mean was 33.4 (range: 4–73 years), The most common HL subtype was nodular sclerosis with 21 cases (37.5%), followed by mixed cellularity with 15 cases (26.8%), lymphocyte-rich with 10 cases (17.9%), lymphocyte-depleted with four (7.1%) cases, and nodular lymphocyte predominant HL (NLPHL) with six cases (10.7%). Patients’ characteristics are detailed in the Supplementary Table 1.

### 3.2 CD161 and LLT1 expression in background immune cells (BICs)

**Figure 1:**
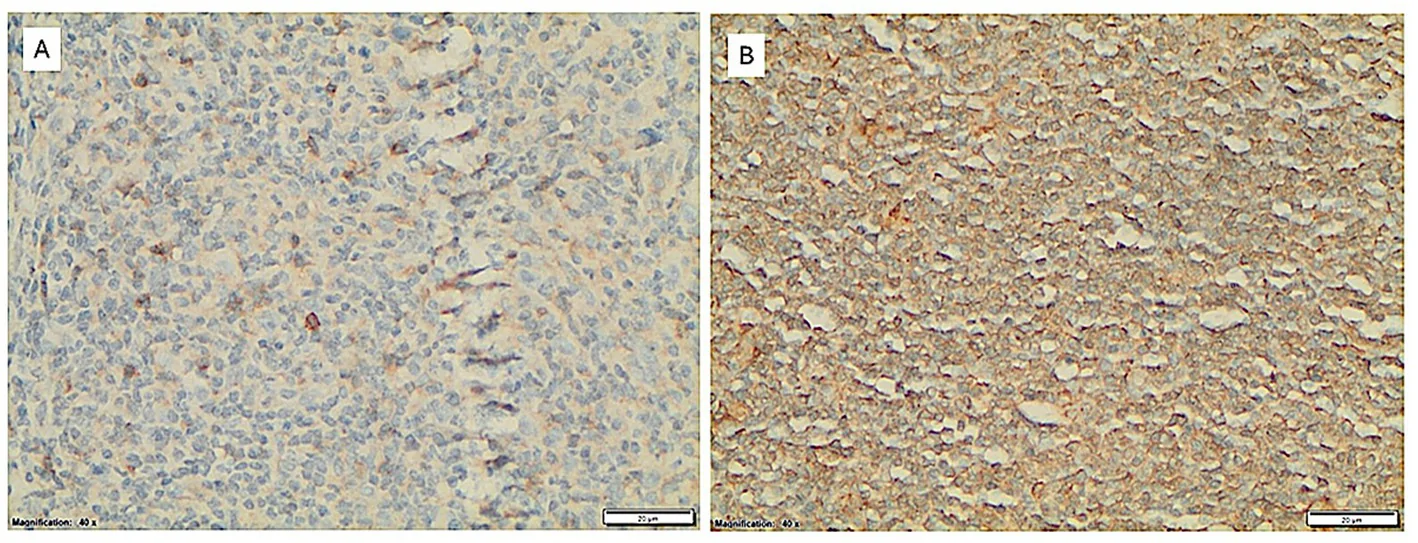
CD161 expression staining in background immune cells (BICs). **(A)** Low-level expression. **(B)** High-level expression. Patients were grouped into low CD161 expression if they had ≤ 50% positive BICs in TME and high CD161 expression if they had > 50% positive BICs in TME (A+B; CD161 IHC,40x). Thirty-three patients (58.9%) were classified in the low (≤ 50% positive cells) frequency group, and 23 patients (41.1%) as high (> 50% positive cells) frequency group.

**Figure 2:**
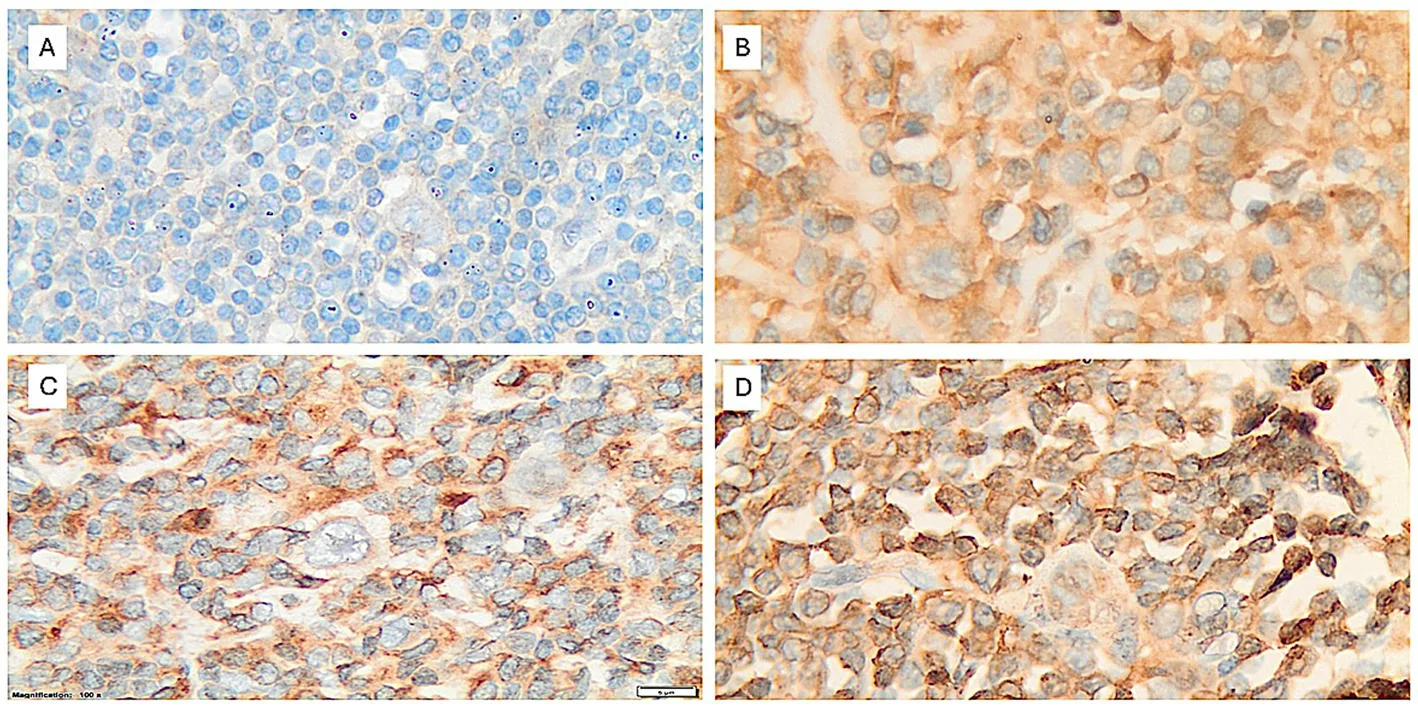
An overview of the observed expression patterns of IHC stain results: CD161 immunohistochemical expression in classical Hodgkin lymphoma **(A)** Negative control showing absence of immunostaining, 40X. **(B)** Background lymphocytes demonstrating cytoplasmic CD161 immunoreactivity, 100X. **(C)** Membranous CD161 expression; Reed–Sternberg (RS) cells (large cell in the center) are negative for CD161 staining, 100X. Panel **C** reproduced from Ref. (26) with attribution under the CC BY License. **(D)** Combined membranous and cytoplasmic CD161 expression, 100X.

Evaluating CD161 expression in HL cases (*n* = 56) (Figure 1), among the 56 patients evaluated, 33 patients (58.9%) were classified as low-frequency group (≤50% positive cells) and 23 patients (41.1%) as high (> 50% positive cells). Membranous-only pattern of expression of CD161 was observed in 12 out of 56 cases (21.4%), cytoplasmic-only in 23 out of 56 cases (41.1%), and 21 cases (37.5%) had both cytoplasmic and membranous (dual positivity). All HL subtypes exhibited CD161-positive BIC infiltration within the tumor microenvironment. Representative immunohistochemical staining patterns of CD161 observed in the study cohort are presented in Figure 2.

**Figure 3:**
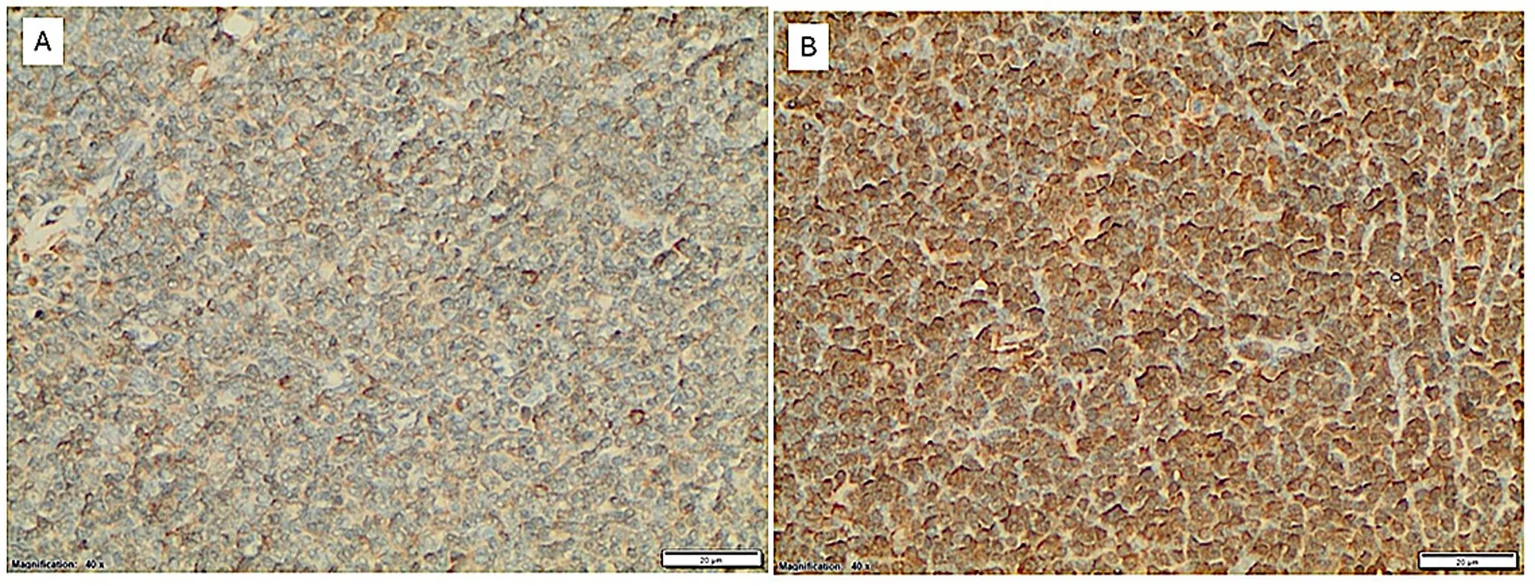
LLT1 expression staining in background immune cells (BICs). **(A)** Low-level expression. **(B)** High-level expression. Patients were grouped into low LLT1 expression if they had ≤ 50%positive BICs in TME and high LLT1 expression if they had >50% positive BICs in TME (A+B; LLT1 IHC, 40x). Six patients (10.7%) had a low (≤50% positive cells) frequency, and a high (> 50% positive cells) frequency was in 50 patients (89.3%).

LLT1 expression in background immune cells (BICs) is shown in Figure 3. LLT1-positive BICs showed a marked predominance of high-frequency expression. Six patients (10.7%) had a low frequency (≤50% positive cells), while 50 patients (89.3%) had a high frequency level (>50% positive cells). Regarding the localization pattern, combined membranous and cytoplasmic staining was the most common, observed in 31 of 56 cases (55.3%), followed by cytoplasmic-only staining in 17 cases (30.4%) and membranous-only staining in 8 cases (14.3%).

### 3.3 Immune cells composition within HL tumor microenvironment

Assessment of immune cell composition within HL tumor microenvironment shows predominance of CD3^+^ T cells. CD3 immunohistochemical stain was available for 34 cases. Mean and median CD3^+^ T-cell frequency of positive immune cells were 62.7% and 70%, respectively. Additionally, 2.9% of the cases (1/34) had a low frequency of positive immune cells, 32.4% (11/34) had a moderate frequency of positive immune cells, and 64.7% (22/34) had a high frequency of positive immune cells.

CD8^+^ cell percentages vary between 5 and 40% of the immune cell population. In our cohort, 60.7% of cases (34/56) show low (≤10% CD8-positive cell) frequency, while 22/56 (39.3%) cases show moderate (>10%–50% CD8^+^ cells), and no cases show high (>50% CD8^+^ cells) frequency. The frequency of CD4^+^ cells was estimated using the available CD3 and CD8 data. The estimated mean and median for CD4^+^ cell frequencies were 86.8% and 90%, respectively.

CD20^+^ B-cell frequency of positive immune cells was assessed in 34 cases. Of which 14.7% (5/34) cases showed low frequency of positive immune cells, 64.7% (22/34) cases had moderate frequency of positive immune cells, and 20.6% (7/34) cases had high frequency of positive immune cells.

Hence, the assessment indicates that the tumor microenvironment in Hodgkin lymphoma is T-cells rich, characterized by abundant CD4^+^ helper T-cells, relatively low CD8^+^ cytotoxic T-cell infiltration, and moderate CD20^+^ B-cell composition.

### 3.4 Statistical correlations between immune cell markers and clinical-pathological characteristics

#### 3.4.1 Correlations of CD161 with LLT1 positive BIC infiltration

**Figure 4:**
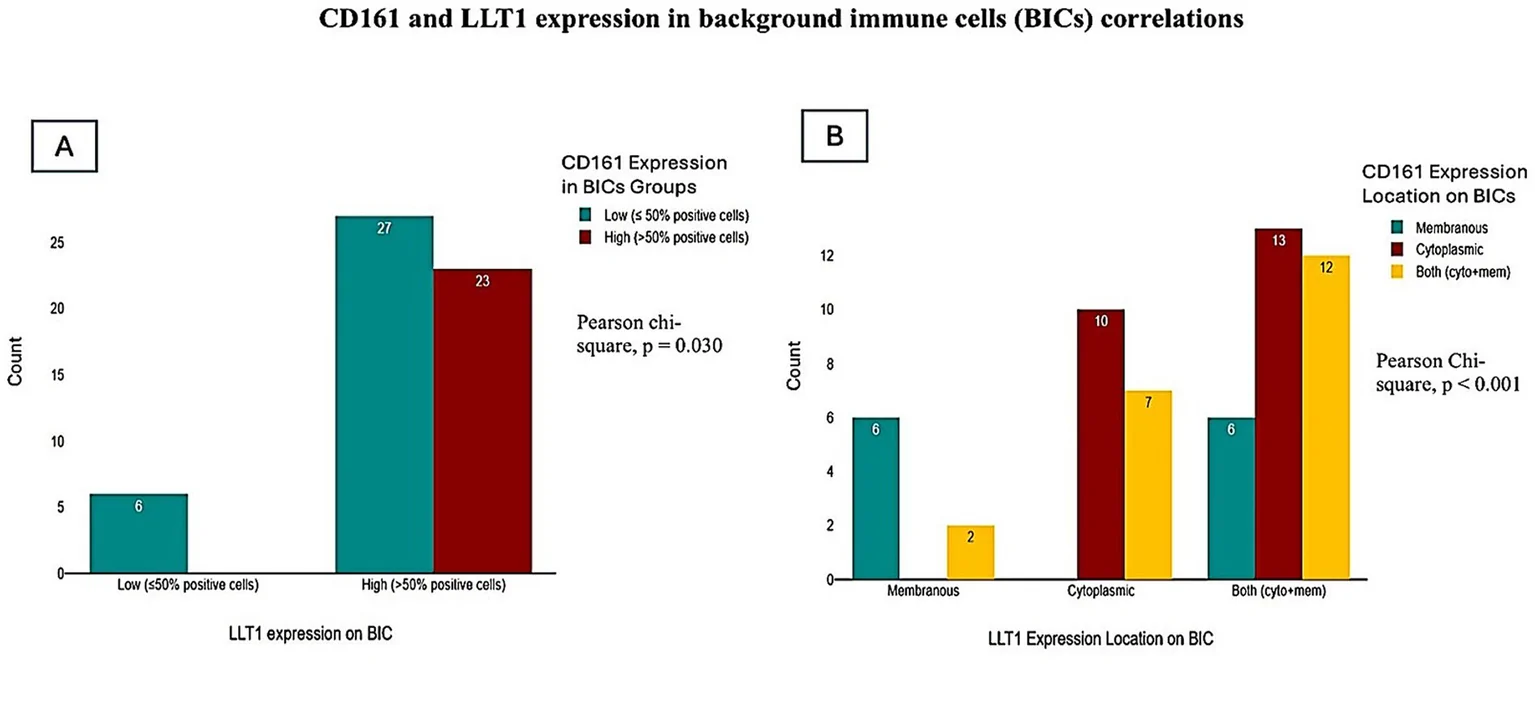
Correlation of CD161 and LLT1 expression in background immune cells (BICs). **(A)** Association between LLT1 and CD161 expression frequencies in BICs (*n* = 56). All cases with low LLT1 expression (≤50% positive cells) exhibited low CD161 expression (*n* = 6). Among cases with high LLT1 expression (>50% positive cells), 27 demonstrated low CD161 expression and 23 demonstrated high CD161 expression. A significant association was observed between LLT1 and CD161 expression frequencies (Pearson’s *χ*^2^ test, *p* = 0.030). **(B)** Correlation between the cellular localization patterns of LLT1 and CD161 expression in BICs (membranous, cytoplasmic, or combined membranous/cytoplasmic; *n* = 56). The localization patterns were strongly associated (Pearson’s χ^2^ test, *p* < 0.001).

After assessing the frequency of CD161 and LLT1 positive cells in the background immune cells (BICs) within the tumor microenvironment (TME), the correlation between their frequency groups were evaluated. A statistically significant association was observed (Pearson chi-square, *p* = 0.030). This finding was confirmed by Fisher’s Exact test (*p* = 0.037) (Figure 4A).

Furthermore, Spearman’s rank correlation demonstrated a weak-to-moderate positive association between CD161 and LLT1 positive BICs frequency groups (*ρ* = 0.289, *p* = 0.031), indicating that higher CD161 infiltration tended to be associated with higher LLT1 infiltration.

To further investigate the potential interaction between CD161 and LLT1, their subcellular localization in BICs was analyzed. A significant association was identified between the localization patterns of CD161 and LLT1 (Pearson Chi-square, *p* < 0.001). Specifically, membranous CD161 expression strongly correlated with membranous LLT1 localization (Figure 4B).

#### 3.4.2 CD161 and LLT1 frequency correlation with EBV status

There was no significant difference between EBV-positive and EBV-negative HL patients in CD161-positive BIC infiltration percentages (*p* = 0.746). The relationship between EBV status and the percentage of LLT1 positive BICs was assessed and showed a statistically significant difference between EBV-positive and EBV-negative patients (*p* = 0.036). The mean rank of LLT1-positive BICs was higher in EBV-negative cases (mean rank = 33.90, *n* = 21) compared to EBV-positive cases (mean rank = 25.26, *n* = 35), indicating that the frequency of LLT1 positive BICs was significantly higher in EBV-negative Hodgkin lymphoma samples.

#### 3.4.3 CD161 and LLT1 positive BIC infiltration correlations with HL subtypes and stages

**Figure 5:**
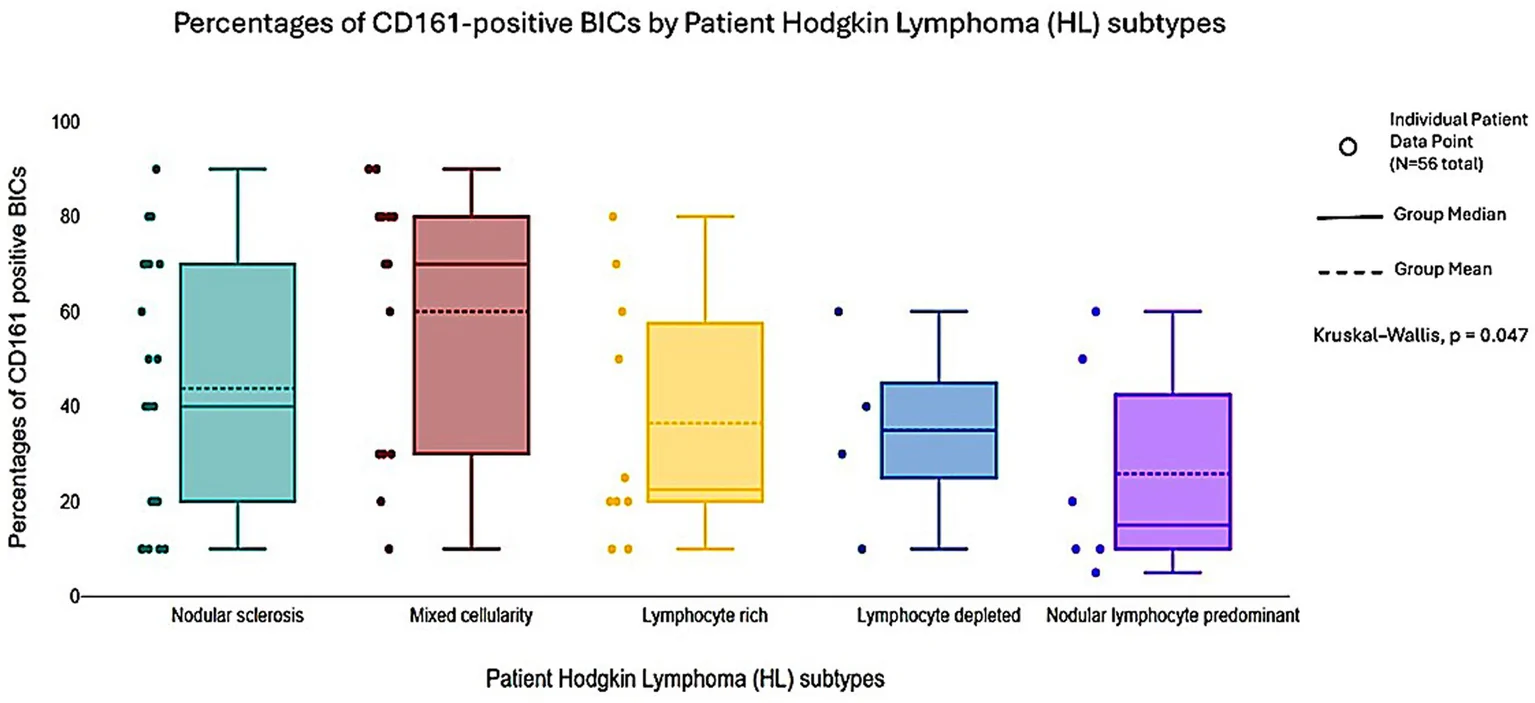
Distribution of CD161-positive BICs percentages among Hodgkin lymphoma subtypes. Comparing CD161 percentages (continuous variable) across the five HL subtypes: Mixed cellularity (*n* = 15), Nodular sclerosis (*n* = 21), Lymphocyte depleted (*n* = 4), Lymphocyte rich (*n* = 10), and Nodular lymphocyte predominant (*n* = 6). The filled circles represent single-patient percentage values (*N* = 56 total). The overall differences across the five subtypes are statistically significant according to the Kruskal–Wallis test (*p* = 0.047).

Studying differences in the percentage of CD161-positive BICs and HL subtypes revealed a statistically significant difference among the five HL subtypes (*p* = 0.047). Mixed cellularity exhibited the highest mean rank, while nodular lymphocyte-predominant subtype had the lowest mean rank of CD161-positive BIC percentage (Figure 5).

**Figure 6:**
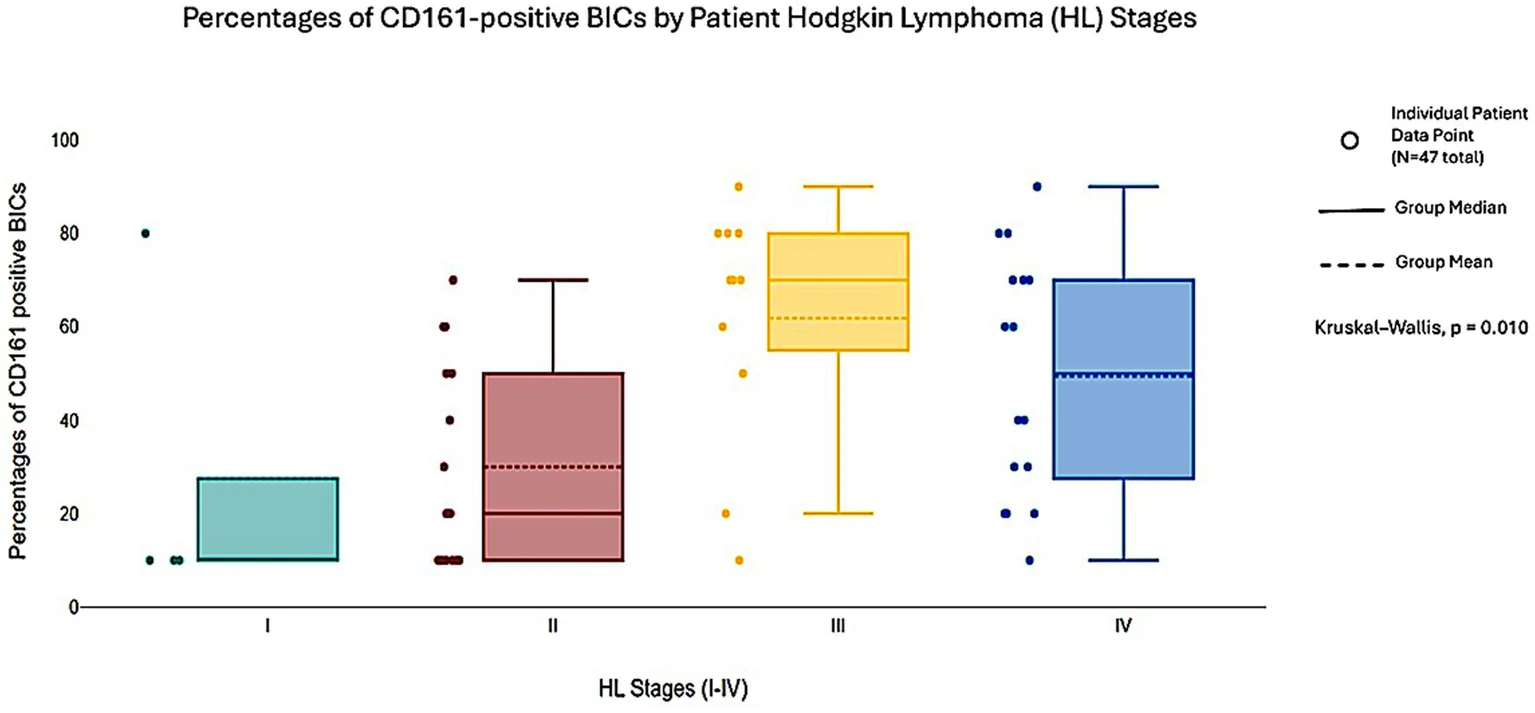
Distribution of CD161-positive BICs percentages among Hodgkin lymphoma stages. Comparing CD161 percentages (continuous variable) across HL stages (I–IV). Stage I (*n* = 4), Stage II (*n* = 16), Stage III (*n* = 11), and Stage IV (*n* = 16). The filled circles represent single patient percentage values (*N* = 47 total). Kruskal–Wallis test: The highest mean rank is observed in stages III and IV and the lowest in stages I and II, *p* = 0.010.

Furthermore, a statistically significant difference was observed among HL stages (*p* = 0.010). HL stages III and IV exhibited the highest mean rank of CD161 frequency (Figure 6), which suggests greater infiltration of CD161-positive immune cells into the TME in advanced stages. While the frequency of LLT1 positive BIC infiltration did not show a significant difference between HL subtype (*p* = 0.720), or HL stages (*p* = 0.250).

#### 3.4.4 Other correlations of CD161 and LLT1 positive background immune cells (BICs) infiltration

No statistically significant or differences were found between the percentages of CD161 positive BICs and clinical-pathological characteristics, including relapses (*p* = 0.387), extranodal involvement (*p* = 0.130), necrosis in tumor microenvironment (*p* = 0.354), or gender (*p* = 0.091). No correlation between percentages of CD161-positive immune cells and other immune cell markers such as CD20 (*p* = 0.525), CD3 (*p* = 0. 981), CD8 (*p* = 0. 206), or CD4 (*p* = 0. 242). Furthermore, no correlation with the patients’ age at diagnosis (*p* = 0. 411).

Additionally, percentages of LLT1-positive BICs did not show significant associations with relapse (*p* = 0.304), extranodal involvement (*p* = 0.856), or gender (*p* = 0.885). No correlation between the percentages of LLT1-positive BICs and immune cell markers, including CD20 (*p* = 0.512), CD3 (*p* = 0.596), CD8 (*p* = 0.763), or CD4 (*p* = 0.885). Furthermore, there is no correlation with the patients’ age at diagnosis (*p* = 0.097). Overall, indicates that the infiltration of LLT1 and CD161 positive BICs in HL TME may be independent of these clinical and immunophenotypic markers.

### 3.5 LLT1 and CD161 expression in BICs and HL patients’ outcomes

#### 3.5.1 CD161 and LLT1 expression in BICs and overall survival (OS)

Previous studies have investigated the effect of CD161 and LLT1 positive BIC infiltration on overall survival (OS) in patients with other types of cancer (19, 20, 27, 28). Thus, we tested their effects on our HL patients’ outcomes.

**Figure 7:**
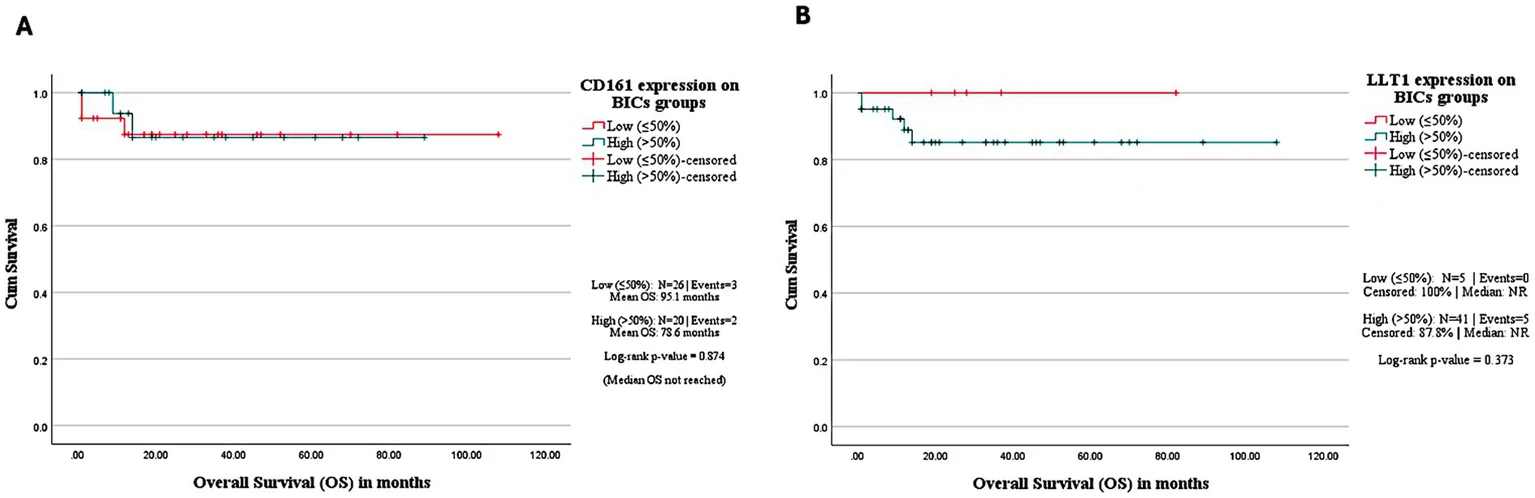
Kaplan–Meier curve of CD161 and LLT1 positive BIC frequency groups and overall survival (OS). **(A)** CD161-positive immune cells infiltration frequency and OS. Patients were grouped according to the percentage of CD161-positive cells IHC scores into low frequency (≤50% positive cells, (*n* = 26), 3 events) and high frequency (>50% positive cells, (*n* = 20), 2 events) groups. No statistically significant difference in OS was observed between the two groups (Log-rank *p* = 0.874). Mean survival times were 95.1 months for the low-frequency group and 78.6 months for the high-frequency group; median survival parameters were not reached due to a high overall censoring rate (89.1%). **(B)** The LLT1-positive immune cells infiltration frequency and OS. Patients were grouped according to LLT1-positive cells percentages IHC scores into low frequency (≤50% positive cells, (*n* = 5), 0 events) and high frequency (>50% positive cells, (*n* = 41), 5 events) groups. No statistically significant difference in OS was observed between the groups (Log-rank *p* = 0.373). The low-frequency group exhibited a 100% censoring rate (0% event rate); median survival times were not reached (NR) due to high censoring (89.1% overall). The steps dropping down on Kaplan–Meier lines represent the deaths (events). The vertical ticks represent the censored cases (patients still alive at that month).

For the survival analysis, follow-up data for OS were available for 46 patients. Analysis revealed no statistically significant difference in OS between patients with low and high CD161 frequency in BICs (Log-rank *p* = 0.874). The low CD161 frequency group (*n* = 26) exhibited a mean survival time of 95.1 months, with three events, while the high CD161 frequency group (*n* = 20) had a mean survival time of 78.6 months, with two events. Median survival times were not reached in either group due to the high rate of (89.1%) censored cases (Figure 7A). Moreover, there was no statistically significant difference in OS among patients with low or high LLT1 frequency groups (Log-rank *p* = 0.373). In the low LLT1 frequency group (*n* = 5), no clinical events occurred (zero% event rate), resulting in a 100% censoring rate. In the high LLT1 frequency group (*n* = 41), a total of five events (12.2%) were recorded. Due to the high rate of overall censoring (89.1%) and the zero event status in the low-frequency group, median survival times could not be reached for either group (Figure 7B).

#### 3.5.2 CD161 and LLT1 frequency within BICs and progression-free survival (PFS)

**Figure 8:**
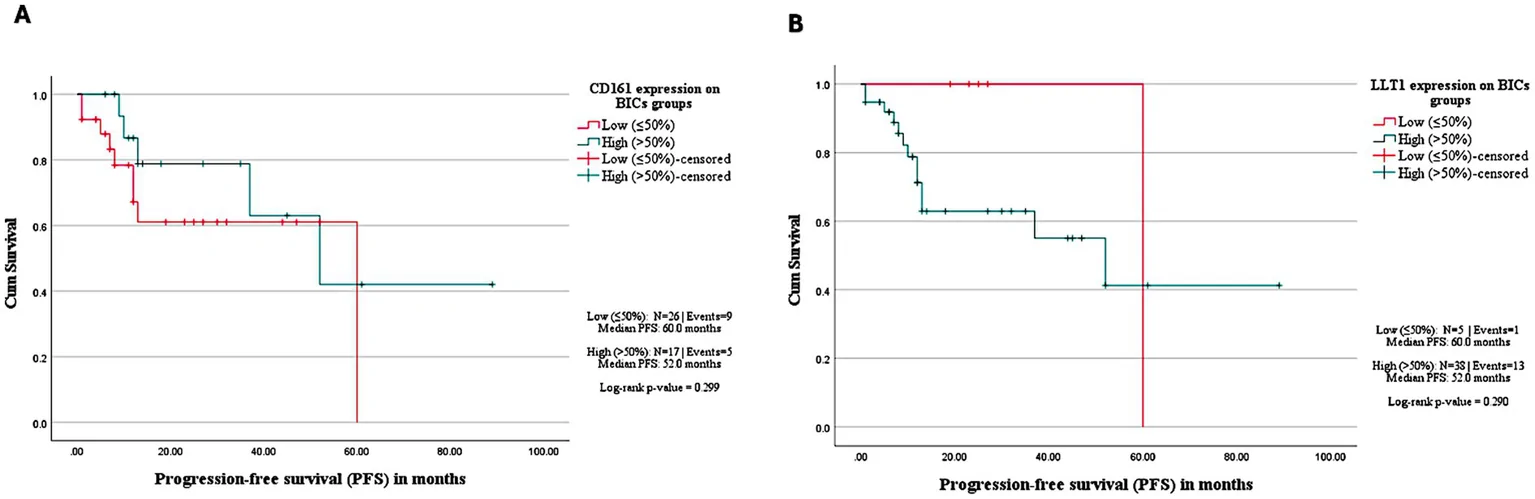
Kaplan–Meier curve of CD161 and LLT1 frequency groups and progression-free survival (PFS). **(A)** The CD161-positive immune cells infiltration frequency and PFS. Patients were grouped according to the percentages of CD161 positive cells IHC scores into low frequency (≤50% positive cells, (*n* = 26), nine events) and high frequency (>50% positive cells, (*n* = 17), five events) groups. The median PFS was 60.0 months for the low frequency group and 52.0 months for the high frequency group. No statistically significant difference in PFS was identified between the two groups (Log-rank *p* = 0.299). **(B)** The LLT1-positive immune cells infiltration frequency and PFS. Patients were grouped according to the percentages of LLT1 positive cells’ IHC scores into low frequency (≤50% positive cells, (*n* = 5), one event) and high frequency (>50% positive cells, (*n* = 38), 13 events) groups. The median PFS was 60.0 months for the low frequency group and 52.0 months for the high frequency group. No statistically significant difference in PFS was observed between the groups (Log-rank *p* = 0.290). The steps dropping down on Kaplan–Meier lines represent the relapsed/deaths (events). Vertical tick marks denote censored observations (patients maintaining complete remission at last follow-up).

To study the association of CD161 and LLT1 frequency in background immune cells (BICs) and progression-free survival (PFS). Follow-up data for PFS analysis were available for 43 patients, during which a total of 14 clinical events (relapse or death) were documented. For the CD161 groups, 14 events occurred across the 43 patients. The low CD161 frequency group (*n* = 26) recorded nine events (34.6% event rate, 65.4% censored), showing a median PFS of 60.0 months. The high CD161 frequency group (*n* = 17) recorded five events (29.4% event rate, 70.6% censored), with a median PFS of 52.0 months. This variation was not statistically significant (Log-rank *p* = 0.299; Figure 8A).

Similarly, LLT1 frequency groups demonstrated no significant association with PFS (Log-rank *p* = 0.290) (Figure 8B). The small low LLT1 frequency group (*n* = 5) had only one event that occurred at month 60, yielding a median PFS of 60.0 months. The high LLT1 frequency group (*n* = 38) had 13 events (34.2% event rate, 65.8% censored), exhibiting a median PFS of 52.0 months; mean: 50.1 months.

The limited cohort size may have restricted the statistical power to detect subtle associations in the survival analyses.

## 4 Discussion

By mass cytometry, Cader et al. (29) showed that HL contains abundant CD4^+^ T cells, including PD-1^+^ exhausted effectors and regulatory cells, along with CD161-expressing NK cells, γδ T cells, and MAIT-like cells. Our study cohort also showed a CD8-poor, CD4-rich TME, supporting an immune-polarizing rather than cytotoxic role for CD161-expressing cells.

A significant correlation was observed between CD161 and LLT1 expression levels and localization. This may suggest that LLT1/CD161 expression on BICs within the HL TME points to a potentially significant role beyond the expression on RS cells and immune cells. Literature has described an interaction between CD161 on BICs and LLT1 on RS cells to provide or facilitate the tumor-escaping mechanism (15, 18, 20, 21). Although our study did not provide an actual investigation to detect the function of the CD161/LLT1 axis interaction, co-expression, and enrichment. But it may suggest that this axis may also have a role in influencing immune regulatory loops, where LLT1-expressing immune cells interact with CD161 on neighboring lymphocytes to modulate activation, cytokine release, and retention.

Zhou et al. (11) found that CD161 (KLRB1) was strongly associated with LLT1 (CLEC2D) proteins, and their membrane localization is essential for direct interaction. They also demonstrated that CD161 expression in different solid tumors correlated with increased expression of T cell exhaustion markers (PD1, TIGIT, LAG3, CTLA4, CD96), tissue-homing markers, and immune-activating and immunosuppressive genes. Thus, the CD161 expression could correlate with immune regulation and infiltration, but also with immune exhaustion and suppression. This dual function of CD161 may explain why it is correlated with better or worse outcomes in cancers.

Such a mechanism aligns with high infiltration of immunosuppressive cell populations in HL TME, which maintains a tightly regulated immune microenvironment and promotes RS cells’ evasion, survival, and proliferation despite having few malignant RS cells (3, 6, 7). Through the release of cytokines, chemokines, and growth factors that influence the polarization of immune cells into an immunosuppressive form that rosette around RS cells (8, 30, 31). Furthermore, these immunosuppressive cells enhance the expression of immunoinhibitory checkpoints (LAG-3, TIGIT, and PD-1) that can promote RS cells’ survival (8).

Thus, it may position the LLT1/CD161 axis in immune-to-immune interaction to facilitate chronic immune modulation, cytokine release, tissue remodeling, and immune polarization, promoting tumor persistence and disease progression rather than effective anti-tumor function (12, 14, 24).

The mixed cellularity (MC) subtype and advanced HL stages (Stages III–IV) showed the highest percentage of CD161-positive BIC infiltration within TME. Iliopoulou et al. demonstrated a significantly increased level of CD161-expressing CD4^+^ T cells in advanced-stage of different cancers, along with their enrichment in tumor sites and malignant effusions (11, 18, 32). Also, Zhou et al. (11) demonstrated that higher CD161 expression levels were observed in advanced stages of stomach adenocarcinoma but lower levels in cancers like invasive breast carcinoma, head and neck squamous cell carcinoma, kidney renal papillary cell carcinoma, and others. In the context of HL, advanced disease stages include widespread endothelial activation and chemokine production across multiple lymph nodes and extranodal involvement, which may cause increased recruitment and retention of CD161-expressing immune cells to tissues (33).

Importantly, Iliopoulou et al. (32) demonstrated that CD161-expressing CD4^+^ T cells acquired immunosuppressive properties upon CD3/CD28 co-stimulation, which was mediated by soluble cytokines, including IL-10, IL-4, and TGF-β. Which may suggest that CD161 expression in HL is associated with an inflammatory and immunosuppressive TME, particularly within a CD4-rich, CD8-poor.

Additionally, HL cytokine milieu abundantly secreted by HRS cells and immune cell populations within the TME, may promote immune polarization, reinforcing and sustaining the expansion and survival of CD161-expressing immune cells. Multiple studies in chronic inflammatory conditions, autoimmune diseases and viral infections have suggested the same theories (34–36). These studies demonstrated that CD161-expressing immune cells are not only recruited to sites of tissue injury but can also actively sustain or increase local inflammation. Through chronic immune activation, cytokine release, and immune polarization, promoting tissue damage, disease persistence, and progression rather than effective anti-disease immunity.

In our HL cohort, enrichment of CD161-positive immune cells in advanced stages, mixed cellularity subtype and significant correlation with LLT1 expression on BICs align with this pattern. Suggesting theory that functional studies are required to determine whether CD161/LLT1 interactions may also occur between infiltrating immune cells, not just between RS cells and immune cells, which may contribute to shaping the TME into a pro-tumor one.

EBV-negative cases exhibited higher LLT1 expression in BICs. In contrast, CD161 expression showed no significant association with EBV status. The EBV effect in the HL TME is more complex, potentially involving regulatory pathways, cytokine profiles, or immune evasion mechanisms. EBV-negative RS cells have a higher mutational burden than EBV-positive, in the absence of viral immune-modulatory proteins such as EBNA1, LMP1, and LMP2A, and may rely more on host checkpoints and secreted chemokines (37).

Lack of association with most clinical and pathological characteristics is still important. It may suggest that the expression of CD161 and LLT1 may be independent of these features and may indicate that their expression is affected by local microenvironment factors.

This is a retrospective observational study that faced several limitations, including the low sample size, lack of actual cell functional assays, and double IHC and multiplex immunofluorescence (IF), which may have reduced the statistical power to detect significant associations and limited the generalization of the findings. Also, prevented find a significant association between CD161 or LLT1 and most clinicopathological characteristics or survival outcomes. Finally, immune cells marker data limited our ability to characterize the TME cell composition and their association with CD161 and LLT1 expression and frequency.

## 5 Conclusion

Regardless of the limitations, our study provides a novel assessment of LLT1 and CD161 expressions in the background immune cells within Hodgkin lymphoma tumor microenvironment. Results considered preliminary and serve as a basis for larger, prospective validation studies using double IHC and multiplex IF assays to determine the immune cell types that express each marker. Moreover, investigate their expression changes during different disease phases (at diagnosis, treatment, remission, and relapse), and studying differences in LLT1 and CD161 expression between circulating and tumor-infiltrating immune cells. Finally, the enrichment of LLT1 and CD161 expressing immune cells in advanced stages and mixed cellularity may suggest their contribution in modulating immune cell function. Therefore, further investigation using functional cell-based assays is recommended. This may identify the interactions and mechanistic roles of the LLT1/CD161 axis within the tumor microenvironment to ultimately determine the LLT1/CD161 axis as a promising immunotherapeutic target.

## Data Availability

The original contributions presented in the study are included in the article/Supplementary material, further inquiries can be directed to the corresponding author.
