# Supplementary material for: CD161 and LLT1 expression in immune cell populations of Hodgkin lymphoma tumor microenvironment
Source: Front Med (Lausanne). 2026 Aug 19;13:1914176. doi: 10.3389/fmed.2026.1914176 (PMC13533724; doi:10.3389/fmed.2026.1914176)
Supplement: Supplementary file 1 [file Table_1.DOCX]

**CD161 and LLT1 Expression in Immune Cell Populations of Hodgkin Lymphoma Tumor Microenvironment**

***Supplementary Material***

**Table 1:** Clinical and Pathological Characteristics of the Study Cohort (1)

| Parameters | Category | Count | Percentages (%) |
| --- | --- | --- | --- |
| Population | Total cohort | 56 | 100.0 |
| Gender | Male | 33 | 58.9 |
|  | Female | 23 | 41.1 |
| Patient HL subtypes | Nodular sclerosis | 21 | 37.5 |
|  | Mixed cellularity | 15 | 26.8 |
|  | Lymphocyte rich | 10 | 17.9 |
|  | Lymphocyte depleted | 4 | 7.1 |
|  | Nodular lymphocyte predominant | 6 | 10.7 |
| Stages | I | 4 | 7.1 |
|  | II | 16 | 28.6 |
|  | III | 11 | 19.6 |
|  | IV | 16 | 28.6 |
|  | N/A | 9 | 16.1 |
| Type of treatment | Chemotherapy | 34 | 60.7 |
|  | Chemotherapy and radiation | 8 | 14.3 |
|  | N/A | 14 | 25.0 |
| Experienced relapsed | No | 29 | 51.8 |
|  | Yes | 14 | 25.0 |
|  | N/A | 13 | 23.2 |
| Extranodal involvement | No | 26 | 46.4 |
|  | Yes | 14 | 25.0 |
|  | N/A | 16 | 28.6 |
| OS status | Alive | 41 | 73.2 |
|  | Died | 5 | 8.9 |
|  | N/A | 10 | 17.9 |
| EBV results | Positive | 35 | 62.5 |
|  | Negative | 21 | 37.5 |

Reference

1.	Rjoop A, Al-Samama’h R, Al-Eitan L. Reed-Sternberg cells express CD161 and lectin-like transcript 1 in Hodgkin lymphoma. Front Med (Lausanne). 2026 Mar 10;13. doi:10.3389/fmed.2026.1754727
